# A novel ligand of the translationally controlled tumor protein (TCTP) identified by virtual drug screening for cancer differentiation therapy

**DOI:** 10.1007/s10637-020-01042-w

**Published:** 2021-01-25

**Authors:** Nicolas Fischer, Ean-Jeong Seo, Sara Abdelfatah, Edmond Fleischer, Anette Klinger, Thomas Efferth

**Affiliations:** 1 Department of Pharmaceutical Biology, Institute for Pharmaceutical and Biomedical Sciences, Johannes Gutenberg University, Staudinger Weg 5, 55128 Mainz, Germany; 2 MicroCombiChem GmbH, Wiesbaden, Germany

**Keywords:** Differentiation therapy, Molecular docking, Precision medicine, Targeted therapy, Virtual drug screening

## Abstract

*Introduction* Differentiation therapy is a promising strategy for cancer treatment. The translationally controlled tumor protein (TCTP) is an encouraging target in this context. By now, this field of research is still at its infancy, which motivated us to perform a large-scale screening for the identification of novel ligands of TCTP. We studied the binding mode and the effect of TCTP blockade on the cell cycle in different cancer cell lines. *Methods* Based on the ZINC-database, we performed virtual screening of 2,556,750 compounds to analyze the binding of small molecules to TCTP. The *in silico* results were confirmed by microscale thermophoresis. The effect of the new ligand molecules was investigated on cancer cell survival, flow cytometric cell cycle analysis and protein expression by Western blotting and co-immunoprecipitation in MOLT-4, MDA-MB-231, SK-OV-3 and MCF-7 cells. *Results* Large-scale virtual screening by PyRx combined with molecular docking by AutoDock4 revealed five candidate compounds. By microscale thermophoresis, ZINC10157406 (6-(4-fluorophenyl)-2-[(8-methoxy-4-methyl-2-quinazolinyl)amino]-4(3H)-pyrimidinone) was identified as TCTP ligand with a K_D_ of 0.87 ± 0.38. ZINC10157406 revealed growth inhibitory effects and caused G0/G1 cell cycle arrest in MOLT-4, SK-OV-3 and MCF-7 cells. ZINC10157406 (2 × IC50) downregulated TCTP expression by 86.70 ± 0.44% and upregulated p53 expression by 177.60 ± 12.46%. We validated ZINC10157406 binding to the p53 interaction site of TCTP and replacing p53 by co-immunoprecipitation. *Discussion* ZINC10157406 was identified as potent ligand of TCTP by *in silico* and *in vitro* methods. The compound bound to TCTP with a considerably higher affinity compared to artesunate as known TCTP inhibitor. We were able to demonstrate the effect of TCTP blockade at the p53 binding site, i.e. expression of TCTP decreased, whereas p53 expression increased. This effect was accompanied by a dose-dependent decrease of CDK2, CDK4, CDK, cyclin D1 and cyclin D3 causing a G0/G1 cell cycle arrest in MOLT-4, SK-OV-3 and MCF-7 cells. Our findings are supposed to stimulate further research on TCTP-specific small molecules for differentiation therapy in oncology.

## Introduction

Due to its high involvement in various biological functions, the translationally controlled tumor protein (TCTP) gained increasing interest since its discovery in 1983 in mouse tumor cells [1, 2]. TCTP is an evolutionary highly conserved protein with functions in cell growth, protein synthesis, cytoskeleton, immune response, development, malignant transformation, tumor reversion, induction of pluripotent stem cells and apoptosis. It interacts with a large number of proteins [3]. A direct interaction with the tumor suppressor p53, has been shown [4, 5]. It is expressed in nearly all tissues, especially in mitotically active tissues [6]. TCTP is also overexpressed in a wide range of cancer types compared to normal tissue indicating a critical role in cancer development [7, 8]. Its potential as prognostic tumor marker has been shown in different cancer types, e.g. ovarian [9] and breast cancer [10] and leukemia [11]. TCTP is recognized as target in differentiation therapy, a strategy that aims at the reactivation of endogenous differentiation programs in cancer and, thus, at the elimination of tumor phenotypes [12]. According to the World Health Organization (WHO), cancer is one of the leading causes of death worldwide. Modern cancer therapy is still dependent on classical chemotherapy with a significant lack in tumor selectivity leading to non-tolerable side effects. Because of these side effects, sufficiently high doses cannot be applied to patients to eradicate all tumor cells in the body. However, non-sufficient tumor cell killing facilitates the development of tumor resistance. Differentiation therapy represents an alternative concept pledging higher tumor selectivity and lower side effects. It aims at restoring cellular differentiation and re-gaining cells into normal cellular programs [13]. TCTP is an attractive target for differentiation cancer therapy [14]. Sertraline, thioridazine [15], buclizine, levomepromazine [16] and artesunate [17] have been identified as inhibitors of TCTP by virtual screening and further *in silico* analyses for the identification of novel inhibitors of TCTP have been performed [18]. Nevertheless, highly efficient and selective TCTP inhibitors have not been identified as yet. Therefore, we performed a focused molecular *in silico* screening of the ZINC database comprising of 2,556,750 chemical compounds and identified ZINC10157406 as potential novel TCTP binding compound. Binding of ZINC10157406 and TCTP was verified by a subsequent molecular docking campaign. *In vitro* studies by microscale thermophoresis verified the *in silico* binding. We evaluated the effect of ZINC10157406 on TCTP expression by Western blotting and immunohistochemistry. Finally, we analyzed the inhibitory effect of ZINC10157406 on cell cycle and tumor growth of MCF-7 and MDA-MB-231 breast cancer, SK-OV-3 ovarian cancer and MOLT-4 leukemia cell lines.

## Materials and methods

### Cell culture

According to the NCI-60 Human Tumor Cell Lines Screen (National Cancer Institute, Rockville, USA), MOLT-4 as leukemia, MCF-7 as breast cancer and SK-OV-3 as ovarian cancer sell line show high TCTP expression levels (http://dtp.cancer.gov). Therefore, we have chosen these three cell lines as suitable models for our investigations. MDA-MB-231 as breast cancer cell line showed low TCTP expression and acted as negative control. We obtained MOLT-4 cells from the German Cancer Research Center (DKFZ, Heidelberg, Germany) and cultured them in complete RPMI 1640 medium supplemented with 2 mM L-glutamine, 10% fetal bovine serum (FBS), penicillin (100 U/mL) and streptomycin (100 µg/mL) (Invitrogen, Darmstadt, Germany). The MCF-7 and MDA-MB-231 cell lines were obtained by the DKFZ, and SK-OV-3 cells were kindly provided by Prof. Dr. Albert Jeltsch (Institute for Biochemistry and Technical Biochemistry, University of Stuttgart, Germany). MCF-7, MDA-MB-231 and SK-OV-3 cells were cultured in complete DMEM containing 4.5 g/L glucose, 4 mM L-glutamine, 10% FBS, 100 U/mL penicillin and 100 µg/mL streptomycin (Invitrogen, Darmstadt, Germany). All cells were grown at 37 °C in a 95% humidified 5% CO_2_ environment and sub-cultured twice per week. Experiments were only conducted with cells in the logarithmic growth phase.

### Virtual screening and molecular docking

We performed virtual screening by the PyRx V0.8 software (http://pyrx.scripps.edu) and molecular docking by AutoDock4 (The Scripps Research Institute, CA, USA), as previously reported by us [19]. The compound library we used was obtained from the ZINC database (http://zinc.docking.org/) with the parameters “3D structure available”, “in-stock”, “anodyne reactivity”, “Reference pH”, “charges: 0”, “Molecular weight: 300–400 Da”, “logP: 0–5” contains 2,556,750 chemical compounds. We used ChemSketch (Advanced Chemistry Development Inc., Toronto, Canada) for the generation of the 2D structures and converted them into 3D structures using Open Babel 2.4.0. The 3D protein solution NMR structure of TCTP was downloaded from the Protein Data Bank (http://www.rcsb.org/pdb, PDB ID: 2HR9). We conducted structurally based virtual screening by the PyRx software using the high-performance supercomputer MOGON (Johannes Gutenberg University, Mainz, Germany). We used the best binding compounds with binding energies below or equal to -9 kcal/mol for further molecular docking with AutoDock4 on MOGON. A grid box with 126 point in x-dimension, 124 points in y-dimension and 100 points in z-dimension with a spacing of 0.570 angstrom was set up for docking. For each cycle, we set the docking parameters to 250 runs and 2,500,000 energy evaluations using the Lackmarian Algorithm. Dockings were repeated twice independently. We obtained the corresponding binding energies and the numbers of conformations in clusters from the docking log file (dlg). For the verification of our *in silico* results, we purchased the five best binding compounds for further *in vitro* studies from ChemDiv (San Diego, USA). Artesunate (Sigma Aldrich, St. Louis, USA) was used as reference.

### Microscale thermophoresis

We used microscale thermophoresis to validate interactions between TCTP and compounds 1–5 as described by us before [20]. Each 50 nM human TCTP were labeled according to the Monolith™ NT.115 Protein Labeling Kit BLUE NHS (NanoTemper Technologies GmbH, Munich, Germany) and titrated with compound 1–5 after 10 min incubating in assay buffer (50 mM Tris buffer (pH 7.6) containing 10 mM MgCl_2_, 150 mM NaCl and 0.05% Tween-20) at room temperature. We used hydrophilic capillaries for blue dye and analyzed fluorescence with 60% LED-power and 40% MST-power at 20 °C by the NanoTemper Monolith™ NT (NanoTemper Technologies GmbH, Munich, Germany). Data was analyzed by the MO affinity software (NanoTemper Technologies GmbH, Munich, Germany). All experiments were conducted at least three times.

### Protein expression and purification

*E. coli* K12 ER2566 harboring plasmid pTCTP01 were grown in LB medium containing ampicillin at 37 °C as previously described [16]. We induced TCTP expression with isopropyl-β-D-thiogalactoside (IPTG) and lysed *E. coli* in cell lysis buffer by sonification. After centrifugation, we conducted chitin-affinity chromatography (Qiagen, Germantown, USA). Confirmation of the isolated protein was carried out by Western Blot analysis using TCTP antibody (Pierce TPT1 Polyclonal Antibody, Thermo Fisher Scientific GmbH, RRID: AB_2552642).

### Growth inhibition assay

We evaluated the *in vitro* response of ZINC10157406 by the resazurin (Promega, Mannheim, Germany) reduction assay to assess the growth inhibition towards MOLT-4, MCF-7, SK-OV-3 and MDA-MB-231 cells as previously described [21]. The fluorescent resazurin is metabolically reduced by living cells to the fluorescent resorufin [22]. 100 µL of MCF-7 (10^4^ cells/ mL), 100 µL of MDA-MB-231 (10^4^ cells/ mL) or SK-OV-3 (10^4^ cells/ mL) cells per well were seeded 24 h prior to treatment in a 96-well-plate. Aliquots of 100 µL MOLT-4 suspension (2 × 10^4^ cells/mL) was seeded per well in a 96-well-plate shortly before treatment. Cells were exposed to ZINC10157406 dissolved in DMSO. After 72 h incubation, 20 µL of a 0.01% (w/v) resazurin solution were added to each well and the plates were incubated at 37 °C for 4 h in the dark. We measured the fluorescence in an Infinite M2000 Pro plate reader (Tecan, Crailsheim, Germany). We used Microsoft Excel for the calculation of a calibration curve by linear regression comparing treated and untreated cells. The IC_50_ value is presented as mean ± standard deviation (SD), we repeated the assay twice with six replicates each.

### Immunohistochemistry

MOLT4 leukemia cells were used for cytospin preparations. Therefore, 100 µL of a 10^6^ cells/ mL suspension (treated with different concentrations of ZINC10157406 and artesunate and incubated in a 37 °C humidified 5% CO_2_ atmosphere for 24 h and were centrifuged at 600 rpm for 10 min to attach them to glass slides. The cells were fixed with cold ethanol. TCTP expression was detected by the UltraVision polymer detection kit (Thermo Fisher Scientific GmbH, Dreieich, Germany) according to the manufacturer’s instructions. Slides were immersed in 3% H_2_O_2_ for 10 min at 25 °C to block endogenous peroxidase activity. Slides were rinsed for 5 min in PBS before non-specific binding was blocked by Ultra Vision Block (Thermo Fisher Scientific GmbH) for 5 min. Primary antibody (Pierce TPT1 Polyclonal Antibody, Thermo Fisher Scientific GmbH, RRID: AB_2552642) was applied (dilution 1:200) and incubated in a humidified chamber at 4 °C overnight. The slides were rinsed for 5 min in PBS before Primary Antibody Amplifier Quanto (Thermo Fisher Scientific GmbH) was applied for 10 min at room temperature. Another PBS-washing step followed before HRP Polymer Quanto (Thermo Fisher Scientific GmbH) was applied for 10 min. The slides were washed for 5 min. In the final staining step, 30 µL diaminobenzidine (DAB) Quanto chromogen (Thermo Fisher Scientific GmbH) was solved in 1 mL DAB Quanto substrate (Thermo Fisher Scientific GmbH) and applied to the slides for 5 min. After washing with PBS for 5 min the cells were counterstained in hemalaun solution (Merck KGaA, Darmstadt, Germany) for 3 min and rinsed again in PBS for 5 min, followed by rinsing in tap water for 10 min. Dehydration was conducted by incubation 2 × 1 min in 70% ethanol, 2 × 1 min 96% ethanol, 2 × 1 min ethanol abs., 2 × 5 min xylol, 1 × 2 min xylol, which was followed by embedding using Entellan (Merck KGaA).

Immunohistochemical analyses were performed by Pannoramic Desk (3DHISTECH Ltd., Budapest, Hungary). Membrane bound and cytoplasmatic expression of TCTP were quantified by Pannoramic Viewer software version 1.15 (3DHISTECH Ltd., Budapest, Hungary). Representative expression values were obtained by the selection of six different areas from each probe.

### Cell cycle analysis by flow cytometry

Twenty-four hours prior to treatment, we seeded 5 × 10^5^ MCF-7, MDA-MB-231 or SK-OV-3 cells in DMEM medium in 6-well plates. Directly before treatment, we seeded 10^6^ MOLT-4 cells in RPMI medium in 6-well plates. We added ZINC10157406, artesunate (as positive control) or DMSO (as solvent control) at varying concentrations and incubated for 24 h (37 °C humidified 5% CO_2_ atmosphere). We fixed the cells with ice-cold ethanol (95%), washed with PBS and stained the cells with 50 µg/mL propidium iodide (PI; Sigma-Aldrich, St. Louis, USA) before fluorescence detection by a BD Accuri C6 Flow Cytometer (BD Biosciences, Heidelberg, Germany). The analysis of the histograms was conducted by the FlowJo software (Celeza, Switzerland). We performed all experiments three times.

###  Protein extraction, SDS-PAGE and Western blotting analysis

MOLT-4 cells were seeded in six-well plates and treated with 0.5 × IC50, IC50 and 2 × IC50 of ZINC10157406. Cells were washed with PBS and harvested in 1.5 mL Eppendorf tubes after 24 h incubation time. The protein fraction was extracted using M-PER™ mammalian protein extraction buffer containing 1% Protease Inhibitor (Thermo Fisher Scientific, Waltham, USA) and 1% Phosphatase Inhibitor (Thermo Fisher Scientific, Waltham, USA) for 30 min shaking at 4 °C. After centrifugation at 14,000 × g for 15 min at 4 °C, the protein concentration was measured by NanoDrop 1000 (PEQLAB, Erlangen, Germany). Aliquot of 30 mg/mL were taken for each sample and 6 × Laemmli buffer (12% SDS, 0.06% bromophenol blue, 47% glycerol, 0.06 M Tris-HCl, pH 6.8) was added at an 1/6 volume of each sample. Proteins were denaturized at 95 °C for 10 min and loaded onto 10% sodium dodecyl sulfate polyacrylamide (SDS) gels. The proteins were transferred on a polyvinylideneflouride membrane (Ruti®-PVDF, Carl Roth, Karlsruhe, Germany) and blocked using 5% BSA/TBS-T (Carl Roth, Karlsruhe, Germany). The membrane was incubated with primary antibodies against TCTP (1:1000; Cell Signaling Technology, Frankfurt, Germany, RRID: AB_10828489), p53 (1:1000; Cell Signaling Technology, Frankfurt, Germany, RRID: AB_10695803), Cyclin D1 (1:1000; Cell Signaling Technology, Frankfurt, Germany, RRID: AB_2259616), Cyclin D3 (1:1000; Cell Signaling Technology, Frankfurt, Germany, RRID: AB_2070801), CDK2 (1:1000; Cell Signaling Technology, Frankfurt, Germany, RRID: AB_2276129), CDK4 (1:1000; Cell Signaling Technology, Frankfurt, Germany, RRID: AB_2631166), CDK6 (1:1500; Cell Signaling Technology, Frankfurt, Germany, RRID: AB_2229289), p21 WAF1/CIP1 (1:500; Cell Signaling Technology, Frankfurt, Germany, RRID: AB_2077850), p18 INK4C (1:500; Cell Signaling Technology, Frankfurt, Germany, RRID: AB_331203) p27kip1 (1:1000; Cell Signaling Technology, Frankfurt, Germany, RRID: AB_2077850) and β-actin (1:2,000; Cell Signaling Technology, Frankfurt, Germany, RRID: AB_2223172) for 12 h at 4 °C. Horseradish peroxidase-linked IgG secondary anti-rabbit or anti-mouse antibodies (1:2,000; Cell Signaling Technology, Frankfurt, Germany, RRID: AB_2099233, AB_330924) were added for 1 h at room temperature. For detection, the membrane was incubated for 5 min with Immobilon™ Classico Western HRP substrate (Merck Millipore, Darmstadt, Germany) under light-exclusion. Fluorescence was visualized and band analysis was conducted by Alpha Innotech FluorChem Q system (Biozym, Oldendorf, Germany).

### Co-immunoprecipitation

Experiments were performed with the Dynabeads® Co-Immunoprecipitation Kit (Thermo Fisher Scientific, Waltham, USA) according to the manufacturer’s instructions. MOLT-4 cells were treated with IC_50_ and 2 × IC_50_ concentrations of ZINC10157406 or DMSO as negative control. Cells were harvested and washed after 24 h incubation time. Lysis was conducted according to the Detergent Lysis Method protocol. 0.5% 1,4-Dithiotreitol (DTT) was added to improve stability in Extraction Buffer. The samples were immunoprecipitated with TCTP polyclonal antibody (Proteintech Europe, Manchester, United Kingdom) coupled to magnetic Dynabeads® M-270 Epoxy. After co-immunoprecipitation, 10% SDS-PAGE, Western blot and chemiluminescence detection blot were performed as explained above. Fluorescence was visualized and band analysis was conducted by Alpha Innotech FluorChem Q system (Biozym, Oldendorf, Germany).

## Results

### Virtual screening and molecular docking

**Fig. 1:**
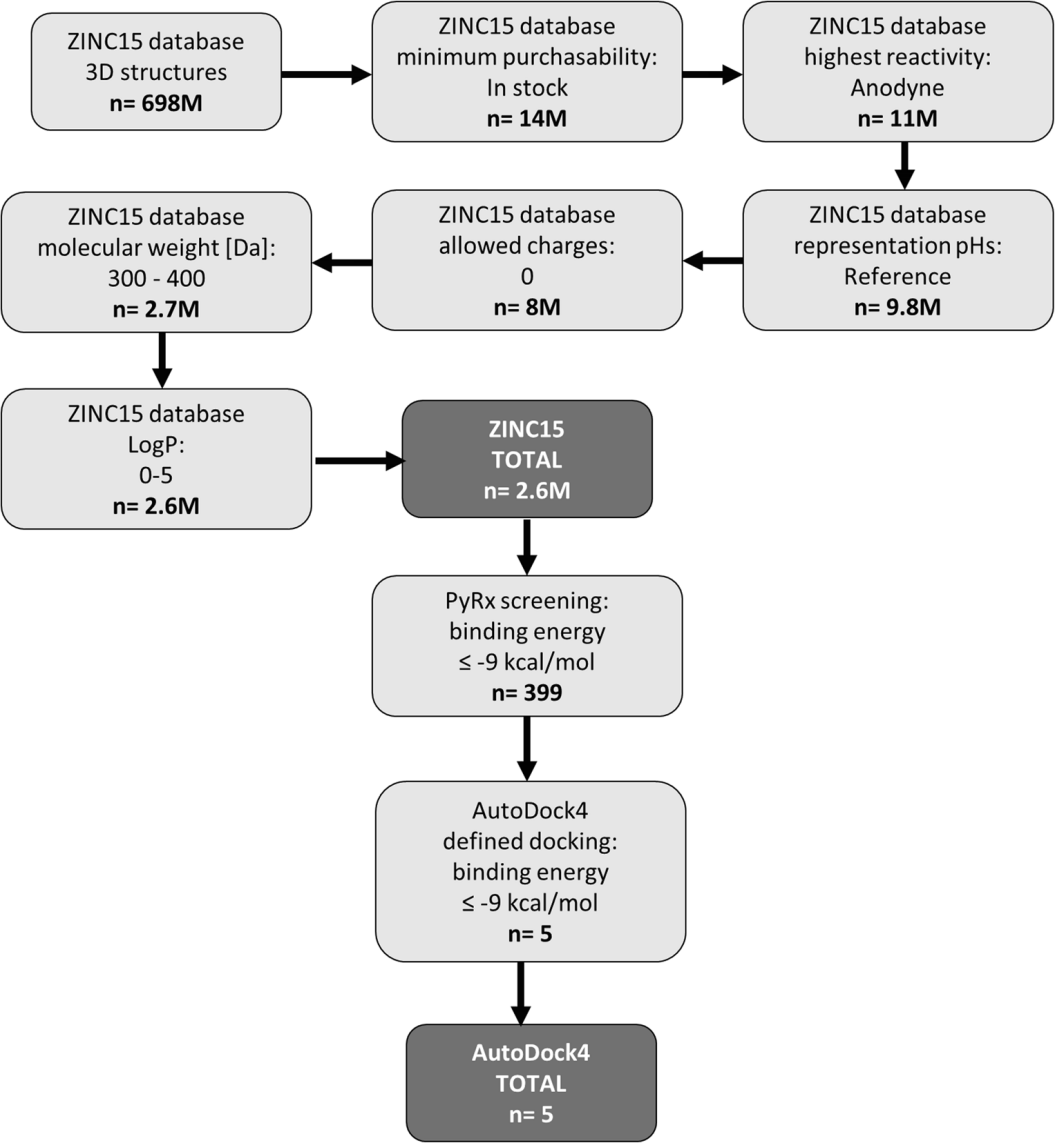
Workflow for screening of the ZINC database

For our studies, we chose a computational approach as efficient way to evaluate the binding of potential chemical binders to TCTP. As first step, we generated our own chemical library derived from the ZINC database (Fig. 1). The ZINC database consists of 697,589,801 3D compounds, of which 13,652,842 were directly available. The filter “anodyne reactivity” removed unsuitable compounds and reduced the total number to 11,215,877 compounds. The pH was limited to “Reference” reducing the number of compounds to 9,639,859. No charges were allowed for an improved computational processability limiting the number of compounds to 7,896,012. The molecular weight was limited to a range between 300 and 400 Da, as most known inhibitors of TCTP are in this range [15, 16]. Of these 2,653,648 compounds, 2,556,750 structures had a logP between 0 and 5 following the Lipinski’s Rule of Five. This set-up database was then used for further *in silico* studies.

**Fig. 2:**
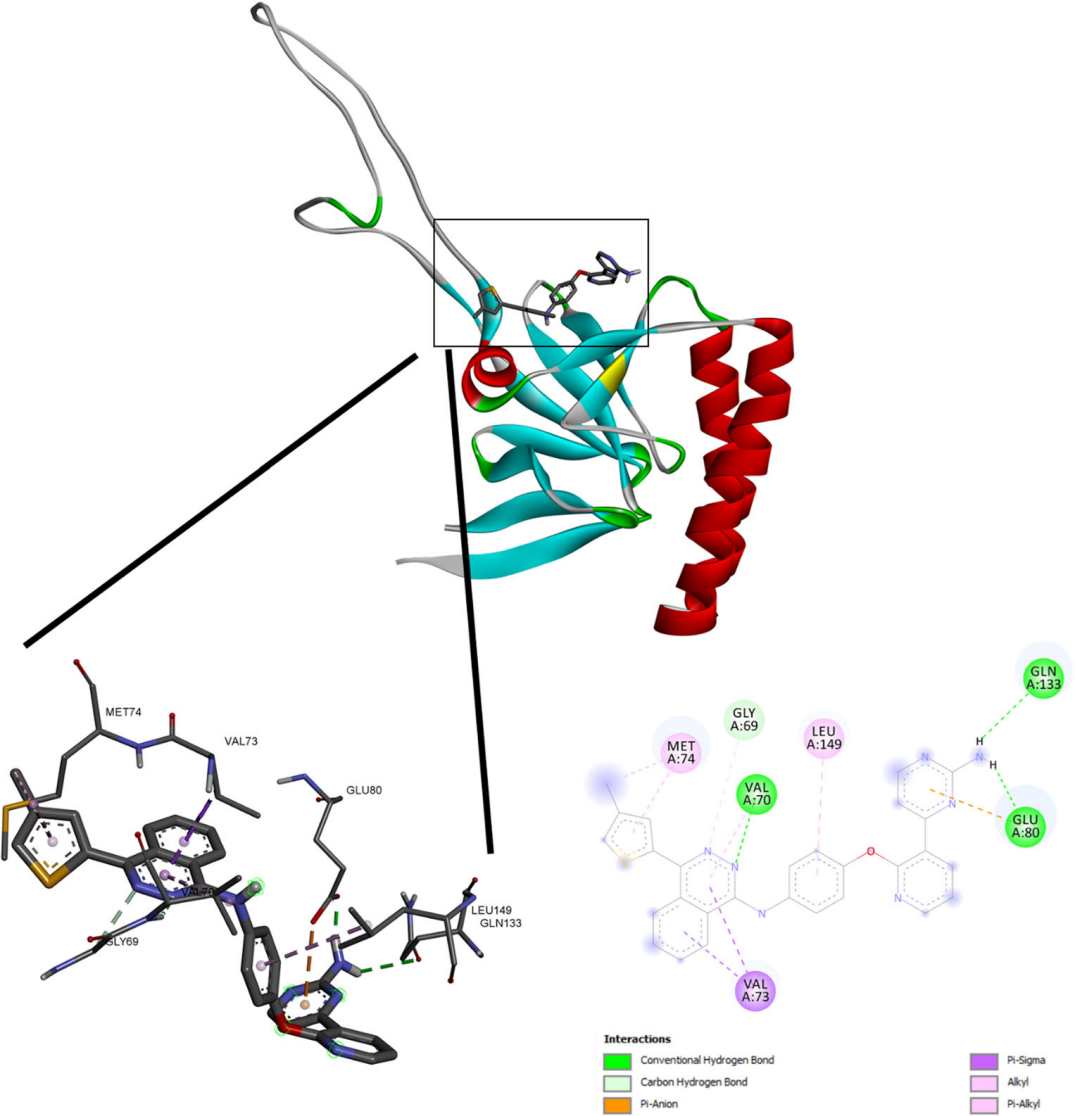
*In silico* binding analysis of ZINC10157406. We used VMD (Visual Molecular Dynamics) for the visualization of the binding pocket

The *in silico* PyRx screening presented 399 compounds with predicted binding affinities ≤ -9 kcal/mol, which were the basis for further AutoDock binding studies. Predicted binding energies and inhibition constants (K_i_) were obtained by *in silico* docking. Five compounds revealed binding energies ≤ -9 kcal/mol (Table 1). ZINC18024062 (2-[(4,6-Dimethyl-2-quinazolinyl)amino]-6-methyl-5-(phenylmethyl)-4(3H)-pyrimidinone) showed the lowest binding affinity (LBE) (-9.77 ± < 0.01 kcal/mol) and the lowest predicted K_i_ (pK_i_) (27.18 ± < 0.01 nM). The LBE of ZINC18158919 (6-phenyl-2-[(4,6,8-trimethylquinazolin-2-yl)amino]-1,4-dihydropyrimidin-4-one) was − 9.76 ± 0.01 kcal/mol and a pK_i_ of 62.45 ± < 0.01 nM. ZIN10157406 (6-(4-fluorophenyl)-2-[(8-methoxy-4-methyl-2-quinazolinyl)amino]-4(3H)-pyrimidinone) revealed an LBE of -9.55 ± 0.00 kcal/mol and a pK_i_ of 79.57 ± 0.02 nM. ZINC17879278 (2-[(4,6-dimethylquinazolin-2-yl)amino]-6-[(pyrimidin-2-ylsulfanyl)methyl]pyrimidin-4-ol) showed a lowest binding energy of -9.50 ± 0.01 kcal/mol and a Ki of 68.29 ± 0.01 nM. ZINC9419129 (2-[(4,6-dimethylquinazolin-2-yl)amino]-6-(3-methoxyphenyl)pyrimidin-4-ol) showed a lowest binding energy of -9.50 ± 0.01 kcal/mol and a Ki of 42.07 ± 0.00 nM. ZINC14096305 (Artesunate) was used as positive control [9, 23–26] and showed a LBE of -7.20 ± 0.00 kcal/ mol and a pK_i_ of 53.67 ± 0.01 µM. Our *in silico* studies indicated that TCTP docked at the p53 binding site [5]. This result speaks for the validity of our molecular dockings, since a TCTP-p53 interaction has been reported before [5]. The binding mode of ZINC10157406 and TCTP is presented in Fig. 2. Val70, Gln1333 and Glu80 are predicted to be involved in hydrogen bonds. Gly69 is expected to be involved in a carbon hydrogen bond. Glu80 is expected to show additional Pi-anion interaction. VAL73 is predicted to make two Pi-Sigma bindings. Met74 and Leu149 are expected to interact hydrophobically.

**Table 1:**
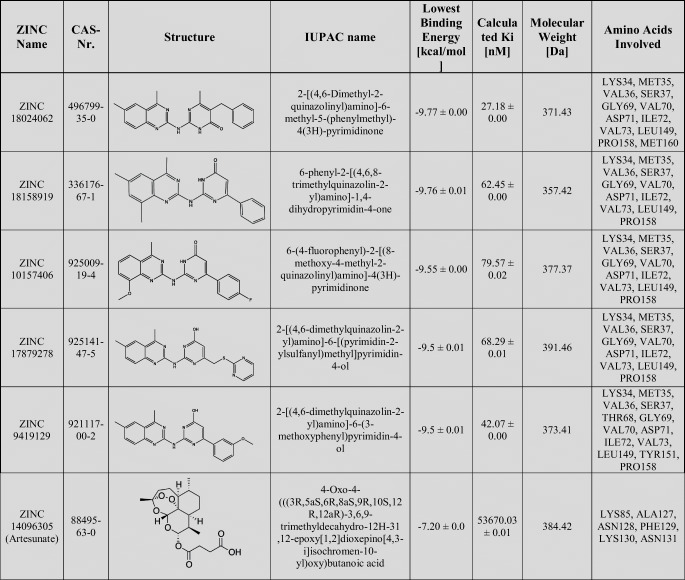
Molecular docking results of the top 5 out of 2.7 million compounds binding to hTCTP and Artesunate as control

### Expression of human seTCTP in *E. coli* K12 ER2566 harboring plasmid pTCTP01

**Fig. 3:**
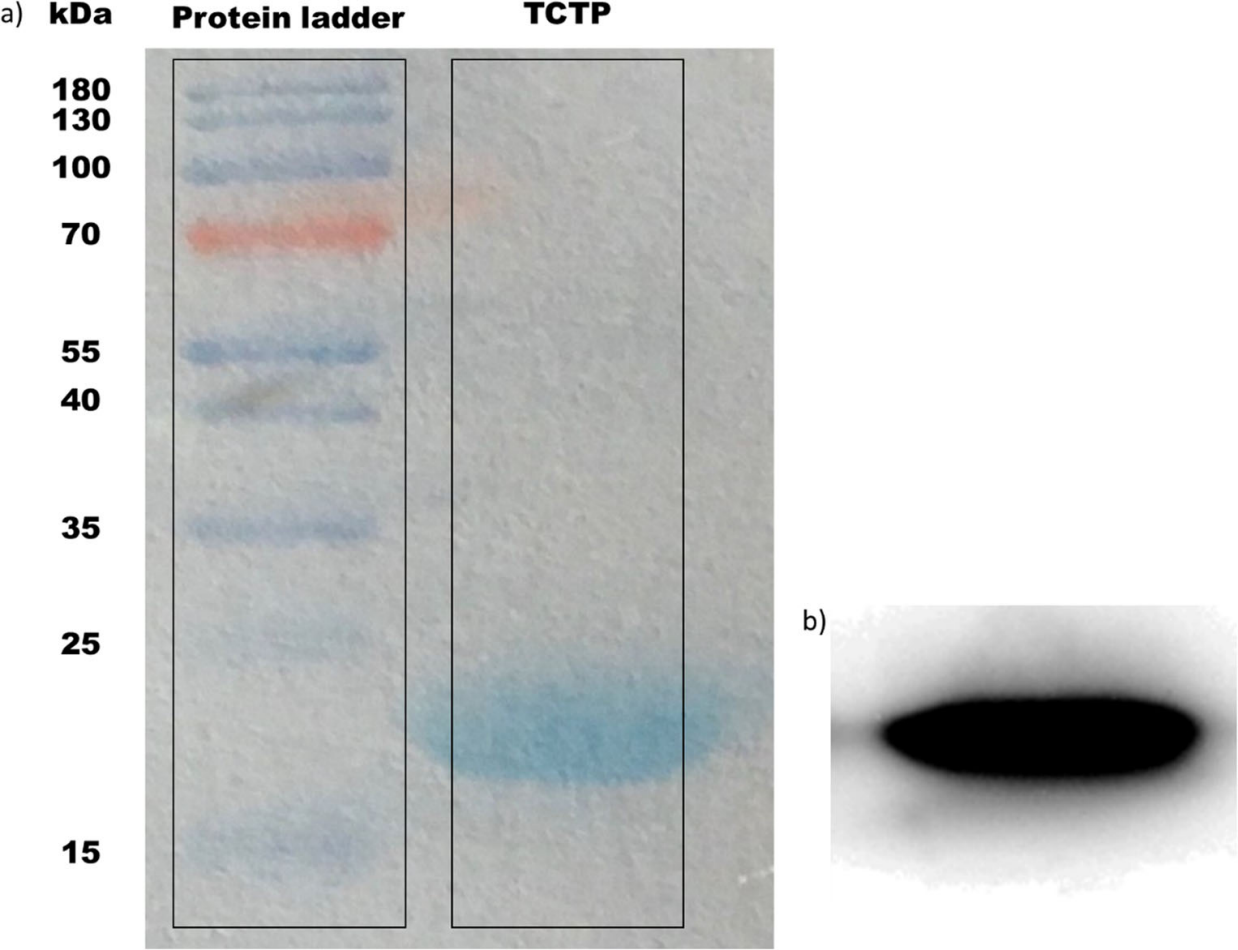
SDS-PAGE of purified hTCTP expressed in *E. coli* K12 ER2566 harboring plasmid pTCTP01 (**a**) and verification by TCTP antibody (**b**)

For microscale thermophoresis, human seTCTP in *E. coli* K12 ER2566 harboring plasmid pTCTP01 was expressed and successfully purified according to a previously described protocol [16]. The expression of seTCTP was demonstrated by SDS-PAGE and verified by Western Blot (Fig. 3). The molecular mass of seTCTP is expected to be between 18 and 20 kDA.

### Microscale thermophoresis

**Fig. 4:**
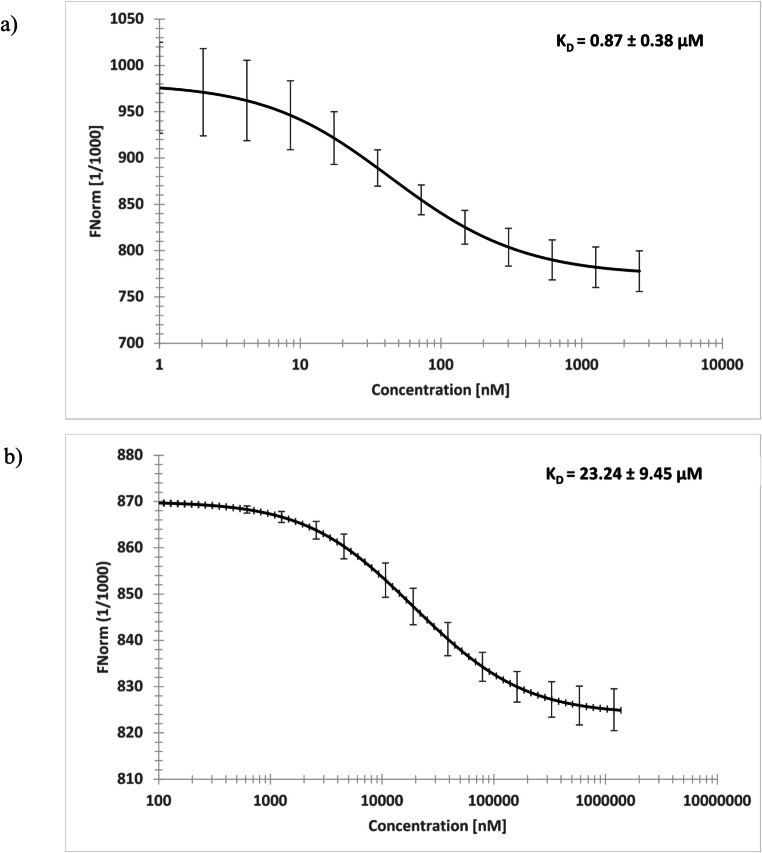
Microscale thermophoresis of human TCTP and ZINC10157406 (**a**) and Artesunate as control (**b**). The experiments were conducted three times independently and values are represented as mean ± standard deviation

Microscale thermophoresis is a highly sensitive technique for the determination of binding affinities between non-labeled molecules and fluorescently labeled target proteins. Labeled human TCTP was titrated with increasing concentrations of compound 1–5. Microscale thermophoresis confirmed our *in silico* screening results. ZIN10157406 showed best *in vitro* binding with a binding affinity of 0.87 ± 0.38 µM. (Fig. 4). Artesunate acted as positive control with a K_D_= 23.24 ± 9.45 µM. Therefore, ZINC10157406 as potential novel TCTP-binding compound was used for further investigations

### Growth inhibition of ZINC10157406 towards SK-OV-3, MCF-7, MOLT-4 and MDA-MB-231 cells

**Fig. 5:**
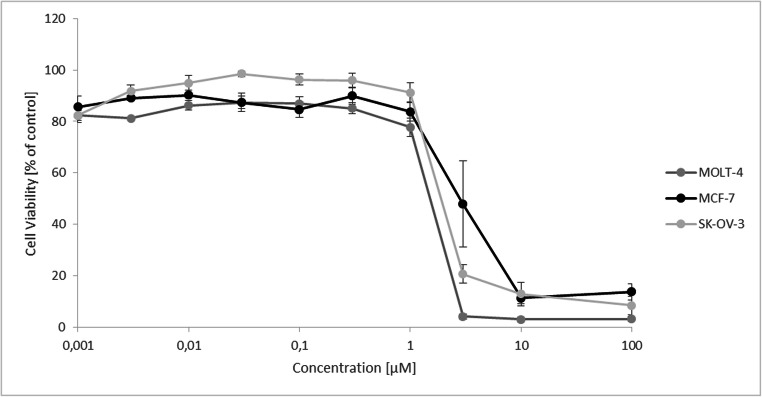
Growth inhibition of MOLT-4, MCF-7 and SK-OV-3 cells by ZINC10157406. Growth inhibition assays were performed three independent times with six measurements each. The curves show the mean values ± standard deviations

Growth inhibitory effects of different concentrations of ZINC10157406 after 72 h incubation time were tested using MOLT-4, MCF-7 and SK-OV-3 cells (Fig. 5). MDA-MB-231 cells were used as negative control (not displayed). ZINC10157406 revealed considerable growth inhibition without apoptosis in all cell lines except MDA-MB-231 cells. The IC_50_ values were 1.91 ± 0.24 µM for SK-OV-3 cells, 3.56 ± 0.77 µM for MCF-7 cells, 1.51 ± 0.08 µM for MOLT-4 cells and above 100 µM for MDA-MB-231 cells. Artesunate as positive control showed IC_50_ values of 20.48 ± 2.73 µM for SK-OV-3 cells, 36.72 ± 8.52 µM for MCF-7 cells and 25.33 ± 2.32 µM for MOLT-4 cells. For MDA-MB-231, no growth inhibitory effect could be observed.

### Western blot detection of TCTP expression in MOLT-4 cells after treatment with ZINC10157406

**Fig. 6:**
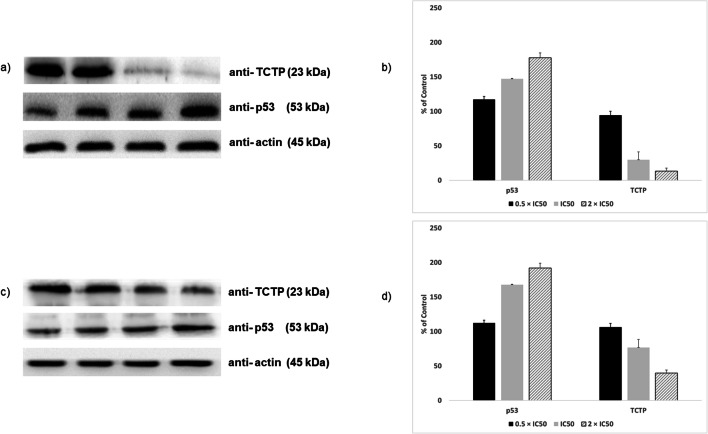
(**a**) Western blot detection of TCTP and p53 expression in MOLT-4 cells after treatment with ZINC10157406 (**a** and **b**) and Artesunate (**c** and **d**). (**a** and **c**) Lane 1: solvent (DMSO), Lane 2: 0.5 × IC50, Lane 3: IC50, Lane 4: 2 × IC50. (**b** and **d**) Quantification of TCTP and p53 expression by ImageJ

After 24 h incubation with ZINC10157406, the TCTP expression significantly decreased by 70.26 ± 6.11% at the IC_50_ concentration and 86.70 ± 0.44% at the concentration of 2 × IC_50_ (*p*˂0.05) (Fig. 6). On the other side, p53 expression increased to 146.99 ± 10.60% after treatment with IC_50_ and 177.60 ± 12.46% after incubation with (2 × IC_50_). Artesunate decreased TCTP expression by 23.19 ± 8.47% (1 × IC_50_) and by 60.25 ± 2.08% (2 × IC_50_). At the same time, p53 expression increased to 167.69 ± 15.77% after application of (IC_50_) of artesunate and 191.87 ± 20.20% after application of 2 × IC_50_ artesunate.

### Analysis of TCTP expression after ZINC10157406 treatment by immunohistochemistry

**Fig. 7:**
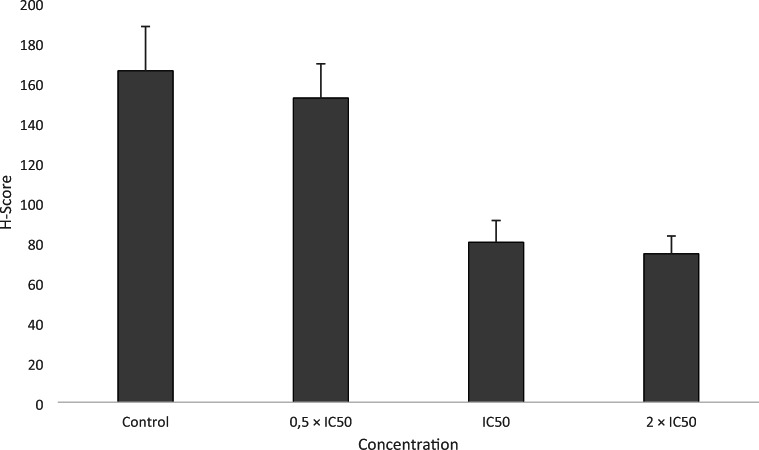
H-score change of immunohistochemically stained MOLT-4 cytospin preparations after 24 h ZINC10157406 treatment at different concentrations. The experiments were conducted thrice

After 24 h incubation with ZINC10157406, MOLT-4 cells were fixed onto a glass slide and incubated with an anti-TCTP antibody. Evaluation of the H-score to quantify the TCTP expression, was conducted by the Pannoramic Viewer software (Fig. 7). Non-treated control MOLT-4 cells showed a H-score of 165.83 ± 22.21, while 0.5 × IC_50_ of ZINC10157406 did not significantly change the H-score (152.25 ± 17.10). However, application of the IC_50_ significantly decreased the H-score to 80.10 ± 10.89 (*p*˂0.05) and application of 2 × IC_50_ decreased the H-score to 74.31 ± 8.85 (*p*˂0.05).

### Cell cycle analysis by flow cytometry

**Fig. 8:**
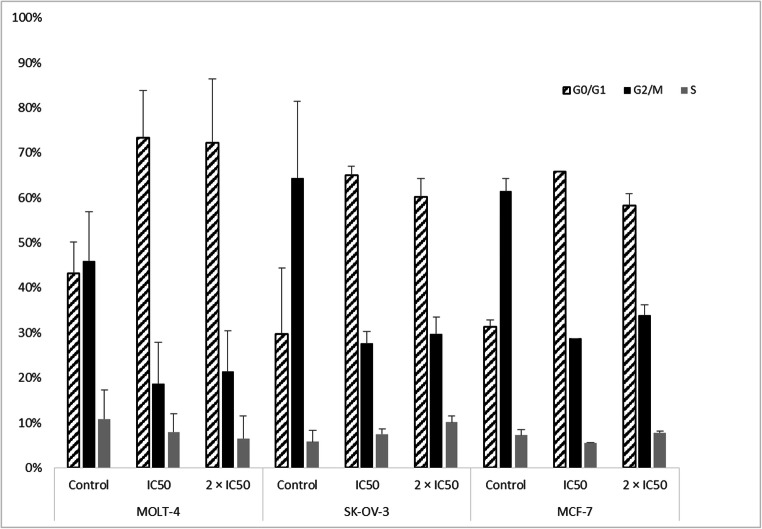
Flow cytrometric analysis of G0/G1 arrest induced by 72 h incubation with ZINC10157406 at IC50 and 2 × IC50. The results represent the mean ± SD of three independent experiments

We evaluated the effect of TCTP-targeting on the cell cycle by flow cytometry. After 24 h incubation with ZINC10157406, cells were analyzed focusing on cells in G0/G1 phase. SK-OV-3, MCF-7 and MOLT-4 cells all showed G0/G1 arrest after treatment with different concentrations of ZINC10157406. DMSO was used as negative control, artesunate was used as positive control. After 24 h of incubation with IC_50_ and 2 × IC_50_ of ZINC10157406, the percentage of cells in G0/G1-phase increased in all three TCTP positive cell lines, whereas the percentage of cells in G2/M-phase decreased as shown in Fig. 8. In MDA-MB-231 cells, concentrations of 10 µM, 20 µM and 40 µM of ZINC10157406 did not show significant changes in the cell cycle (not displayed).

### Cell cycle regulatory protein expression after ZINC10157406 treatment

**Fig. 9:**
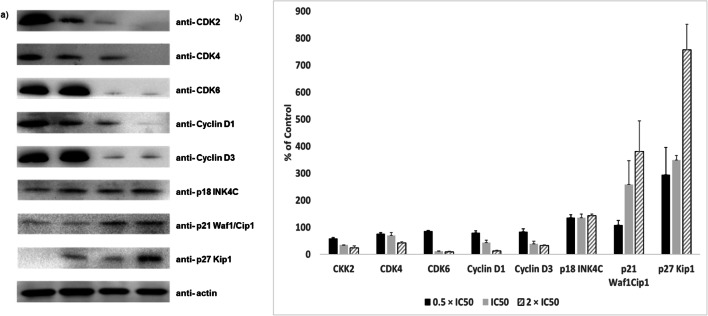
Cell cycle regulatory protein expression after 24 h ZINC10157406 treatment. Lane 1: solvent (DMSO), Lane 2: 0.5 × IC50, Lane 3: IC50, Lane 4: 2 × IC50. Quantification of protein expression (b and d) by ImageJ. All experiments were performed three times

The flow cytometric results were confirmed by Western blot analyses of common cell cycle regulatory proteins that are involved in G1 cell cycle progression [27–29], among them CDK2, CDK4, CDK6, cyclin D1, cyclin D3 and cyclin-dependent kinase inhibitors, p27 and p21. After 24 h incubation with 0.5 × IC_50_, IC_50_ and 2 × IC_50_ of ZINC10157406, the expression of CDK2, CDK4, CDK6, cyclin D1 and cyclin D3 decreased in a concentration-dependent manner, whereas the expression of p18, p21 and p27 increased also in a concentration-dependent fashion as shown in Fig. 9.

### Binding analysis of TCTP and p53 after ZINC10157406 treatment by co-immunoprecipitation

**Fig. 10:**
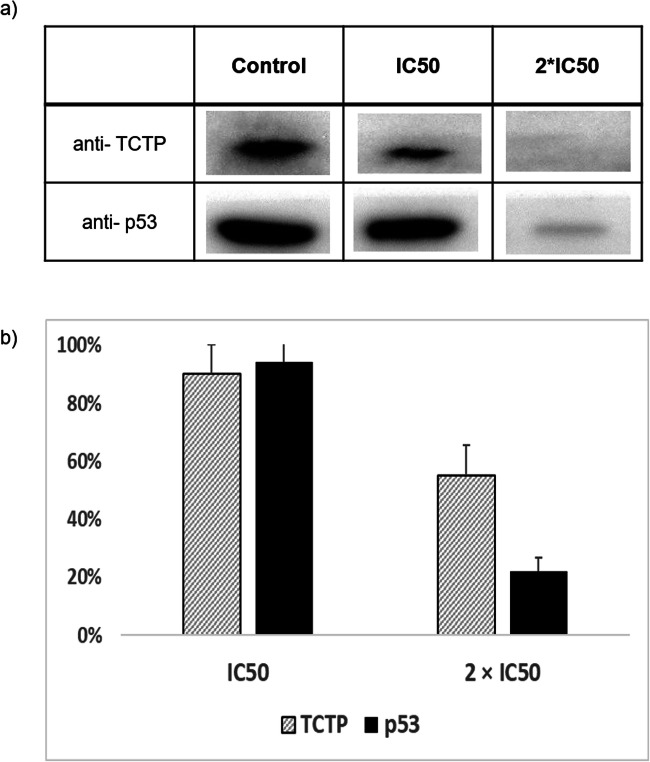
The expression of TCTP and TCTP-bound p53 after ZINC10157406 treatment for 24 h. Quantification of protein expression (b) by ImageJ. The results represent the mean ± SD of three independent experiments

MOLT-4 cells were incubated with ZINC10157406 for 24 h, and TCTP and TCTP-bound p53 were immunoprecipitated. TCTP expression decreased in a concentration-dependent manner. The expression of TCTP-bound p53 decreased even more than TCTP. Increasing the concentration of ZINC10157406 from IC_50_ to 2 × IC_50_ decreased TCTP expression by 35.33 ± 6.92%, whereas TCTP-bound p53 expression decreased by 72.35 ± 3.45% under the same conditions. DMSO as negative control did not influence the expression of TCTP and thus of TCTP-bound p53. Artesunate as positive control also decreased TCTP expression but to a lesser extent i.e. by 12.15 ± 9.97% and expression of TCTP-bound p53 by 36.93 ± 13.28%. The results are displayed in Fig. 10.

## Discussion

In early stages of modern drug discovery, the main pillars are experimental high-throughput screening and virtual screening as theoretical complementary approaches [30–33]. In our study, we successfully used *in silico* methods for the identification of a novel ligand of TCTP as a new target for cancer therapy. TCTP is a highly conserved protein that can be found in all human tissues [34]. High TCTP expression has been linked to cancer [15, 35]. In tumor reversion, TCTP was the most strongly downregulated protein in revertant cells. Lower expression of TCTP resulted either in the reprogramming of cancer cells or apoptosis [36]. Differential expression of proteins or an altered biological function are the fundamental bases of an effective target in cancer therapy. Although survival rates in cancer are increasing [37], it is still one of the main causes of death worldwide. By now, classical chemotherapy is still a mainstay of modern cancer therapy and part of national cancer treatment guidelines all over the world. Despite the fact that chemotherapy has further been developed and selectivity have been improved, it is still accompanied by serious side effects, which limit the therapeutic success [38]. Due to aging populations the impact of cancer as cause of death is expected to rise increasing the urge of finding new therapeutic targets and approaches. Differentiation therapy has been found to be a promising way of a new and effective tumor therapy [39]. Although TCTP represents a suitable target here [40], efficient and selective inhibitors are still lacking.

Our motivation was to conduct a large-scale computational PyRx screening of 2.6 million substances of the ZINC database. We limited the number of compounds due to parameters that matched common drug-like criteria in order focus on promising compounds or lead structures. Our *in silico* screening revealed 399 suitable compounds, of which 5 had binding energies ≤ -9 kcal/mol calculated by molecular docking by AutoDock4. Docking showed binding of those substances at the TCTP-p53 interaction site [5].

Microscale thermophoresis is a highly sensitive technique [41] that showed binding between fluorescently labeled TCTP and ZINC10157406 with a binding affinity of 0.87 ± 0.38 µM, which is to the best of our knowledge by now the first nanomolar binding affinity measured for small molecule TCTP interaction. Before, only interaction between TCTP and antidepressants [15], antihistaminics [16] and artemisinin-derivatives such as artesunate [23] have been reported.

For our research, we conducted the experiments with MOLT-4-, MCF-7 and SK-OV-3 cells, which have a high TCTP expression according to the NCI-60 Human Tumor Cell Lines Screen (http://dtp.cancer.gov), and MDA-MB-231 cells, which have a low TCTP expression, as negative control. ZINC10157406 exerted growth inhibitory effects in all TCTP-positive cell lines after 72 h. For MOLT-4, MCF-7, and SK-OV-3, the IC_50_ values were about 20 times lower than of artesunate as known TCTP inhibitor. In MDA-MB-231 cells, no significant effect could be observed. ZINC10157406 decreased TCTP expression in MOLT-4 cells nearly three times more than artesunate. At the same time, p53 expression increased significantly (*p*˂0.05) if ZINC10157406 was applied. Artesunate as control decreased TCTP expression less strongly. Artesunate itself increased p53 expression, which explains this strong change [42]. We confirmed the decrease in TCTP expression of MOLT-4 cells by immunohistochemical analyses.

These results indicate for a connection between TCTP and p53 and verify our *in silico* results showing a replacement of p53 by our compound at the TCTP binding site. Binding of ZINC10157406 to TCTP was predicted at the p53 binding site, which has been reported before [5].The results speak for a negative feedback loop between TCTP and p53. TCTP promoted p53 degradation, whereas p53 directly repressed TCTP transcription [4].

Blockade of TCTP increased the number of MOLT-4, MCF-7 and SK-OV-3 cells in the G0/G1 phase of the cell cycle a concentration-dependent manner (Fig. 8), whereas no effect could be observed in MDA-MB-231 cells. G1 arrest implies an involvement of p53, which stresses our *in silico* results [43]. This observation strongly suggested that ZINC10157406 caused growth inhibition by G0/G1 cell cycle arrest. In addition, we investigated the expression of cell cycle-dependent proteins, such as CDK2, CDK4, CDK6, cyclin D1 and cyclin D3, which are involved in G1/S progression in mammalian cells [44]. Cyclin-dependent kinases are activated by forming complexes with cyclins and being phosphorylated by CDK-activating kinases [44]. The expression of CDK2, CDK4, CDK, cyclin D1 and cyclin D3 decreased in MOLT-4 cells in a concentration-dependent manner (Fig. 9) indicating a G0/G1 cell cycle arrest. On the other hand, the expression of the CDK inhibitors p18, p21 and p27 [45] increased in a dose-dependent way (Fig. 9) stressing their involvement in ZINC10157406-mediated inhibition of MOLT-4 cell growth.

For additional verification of binding of ZINC10157406 to the TCTP-p53 interaction site, we conducted a co-immunoprecipitation experiment. This is a powerful standard technique for the detection of highly sensitive protein-protein interactions [46, 47]. TCTP dissolved in protein solution derived from lysed MOLT-4 cells upon ZINC10157406 treatment was magnetically precipitated and subjected to Western blot detection. Indeed, TCTP and the p53 fraction bound to TCTP were detected. Thus, we confirmed our hypothesis that ZINC10157406 strongly binds to TCTP and thereby blocks p53 binding. Application of 2 × IC_50_ of the compound to MOLT-4 cells significantly (*p* ˂ 0.05) decreased the TCTP expression but p53 decreased even stronger confirming that the p53 binding site of TCTP was blocked by ZINC10157406. Although still statistically significant, the effects seen with artesunate as positive control were weaker than with ZINC10157406.

In conclusion, we were able to identify ZINC10157406 *in silico* and *in vitro* as novel ligand of TCTP. Our molecular docking results *in silico* were confirmed by flow cytometric studies, Western blotting, immunocytochemistry and co-immunoprecipitation. Hence, we were able to demonstrate that the interaction between TCTP and p53 can be disturbed by specific small molecule ligands. ZINC10157406 is a potent TCTP ligand and may act as valuable lead compound for further drug development with TCTP as treatment target in tumor reversion and cancer therapy.

## Data Availability

The datasets used and/or analyzed during the current study are available from the corresponding author on reasonable request.

## Abbreviations

BSA: Bovine Serum Albumin
CDK: Cyclin Dependent Kinase
CO_2_: Carbon Dioxide
DAB: Diaminobenzidine
DKFZ: German Cancer Research Center
DMEM: Dulbecco’s Modified Eagle’s Medium
DMSO: Dimethyl Sufloxide
DTT: 1,4-Dithiotreitol
FBS: Fetal Bovine Serum
H_2_O_2_: Hydrogen Peroxide
HRP: Horseradish Peroxidase
IC50: Half Maximal Inhibitory Concentration
IPTG: Isopropyl-β-D-thiogalactoside
K_D_: Dissociation Constant
K_i_: Inhibition Constant
LBE: Lowest Binding Energy
LB medium: Lysogeny Broth Medium
MgCl_2_: Magnesium Chloride
NCI: National Cancer Institute
PAGE: Polyacrylamide Gel Electrophoresis
PBS: Phosphate Buffered Saline
PDB: Protein Data Bank
PI: Propidium Iodide
PVDF: Polyvinylideneflouride
RPMI: Roswell Park Memorial Institute
SD: Standard Deviation
SDS: Sodium Dodecyl Sulfate
TCTP: Translationally Controlled Tumor Protein
WHO: World Health Organization
